# Substance abuse disorder in patients with ADHD taking methylphenidate

**DOI:** 10.1192/j.eurpsy.2026.10878

**Published:** 2026-07-31

**Authors:** T. Gutierrez Higueras

**Affiliations:** Child and Adolescent Psyciatry, Servicio Andaluz de Salud, Jaén, Spain

## Abstract

**Introduction:**

Attention deficit hyperactivity disorder (ADHD) is a neurodevelopmental disorder primarily diagnosed in childhood and adolescence and typically treated with methylphenidate (MPH), a stimulant medication effective in managing the core symptoms of inattention and hyperactivity. Treatment with MPH may result in an increased risk of substance misuse and abuse related to the reward and motivational pathway. Understanding the prevalence of substance use, the main substances of abuse, and the clinical consequences in this population is critical to improving treatment strategies and minimizing adverse effects.

**Objectives:**

To evaluate the prevalence and characteristics of substance use patterns in patients with ADHD treated with methylphenidate, as well as to identify the most commonly used drugs and analyze the associated clinical and psychosocial consequences.

**Methods:**

A literature search was conducted in Pubmed examining substance use in patients with ADHD treated with MPH, considering the presence of concomitant psychiatric disorders (mainly substance abuse disorders) and the clinical impact of MPH abuse.

**Results:**

The review indicates that patients with ADHD and psychiatric comorbidities, such as conduct, mood, anxiety, or personality disorders, are at increased risk for MPH misuse. The risk is increased in those with coexisting substance use disorders, with alcohol, cannabis, nicotine, and cocaine use being more common. MPH abuse can generally result in self-administered use of more than prescribed doses or unconventional routes of administration, such as inhalation. The most significant adverse effects include gastrointestinal and cardiovascular events, psychosis, and exacerbation of psychiatric symptoms.

**Conclusions:**

Substance use and abuse are generally common in patients with ADHD, with cannabis, alcohol, and nicotine being the most commonly used drugs. Treatment with methylphenidate does not appear to substantially increase the overall risk of use, although it may be associated with isolated cases of stimulant abuse.

**Disclosure of Interest:**

None Declared

